# Is Being Funny a Useful Policy? How Local Governments’ Humorous Crisis Response Strategies and Crisis Responsibilities Influence Trust, Emotions, and Behavioral Intentions

**DOI:** 10.1007/s13753-022-00436-z

**Published:** 2022-09-09

**Authors:** Janna Hämpke, Stefan Röseler, Meinald T. Thielsch

**Affiliations:** Department of Psychology, University of Münster, 48149 Münster, Germany

**Keywords:** Affect, Crisis communication, Crisis responsibility, Humor, Situational crisis communication theory, Social media

## Abstract

This study is the first to investigate how a local government’s humorously framed response strategy on social media to a low-severity crisis influences people’s trust in the local government and their crisis-related behavioral intentions, specifically when considering the government’s responsibility for the crisis. Based on the situational crisis communication theory, we examined the mediating role of experienced positive or negative affect on people’s responses to a local government’s crisis communication strategy. Further, we exploratorily examined the predictive power and moderating role of demographics, sense of humor, disposition to trust, and the respective crisis scenarios. A total of 517 people participated in an online experiment in which they were confronted with three randomly presented fictive crisis scenarios where the local government’s crisis responsibility (high versus low) and the framing of their crisis response strategy (in form of humorous versus rational Twitter posts) were systematically varied between subjects. First, the results mostly corroborate earlier findings about the degree of crisis responsibility (that is, when a government’s crisis responsibility is high, people have less trust and behavioral intentions) and about the mediating role of experienced affect. Second, we found that humorously framed strategies negatively influence trust and positive affect (but not behavioral intentions). In contrast to earlier findings, the crisis responsibility × framing interaction was not significant. Altogether, the results advise against using humor in crisis communications on social media, even in low-severity crisis. Exploratory analyses indicate that further investigations should focus on specific crisis characteristics and potential moderators.

## Introduction

In crisis situations, social media offers local governments a way to communicate directly and quickly with the public and provide up-to-date information. Such crisis communication allows local governments to stay in contact with the public during a crisis, gain their trust, and encourage them to take appropriate actions (Bakker et al. 2018). Prior research indicates that crisis managers might effectively use humor in social media communications during low-severity crises, such as misconducts or scandals (Avidar 2012), as humorous messages during such crises generally reach more people and are more popular (Choudhary et al. 2012; Fraustino and Ma 2015). Even local governments and public authorities have increasingly begun using less formal and even humorous language on social media (Fraustino and Ma 2015; Rasmussen 2017). For instance, the NSW Australian Police is famous for using humor to communicate public safety messages (NSW Police Force n.d.).

However, research and practical examples indicate that humorous crisis response strategies do not always result in positive responses (Honisch and Más Manchón 2020). For instance, the brand Tropicana missed the mark when responding to the COVID-19 pandemic in a humorous way (Tropicana 2020). For local governments, such misuse can have severe consequences: Inappropriate humor may threaten citizens’ trust in the local government and discourage them from taking appropriate actions (Bitterly 2022). Hence, it is necessary to understand the conditions under which humorous crisis communication on social media might be useful. We contribute to this topic by examining how a local government’s humorous versus rational communications, depending on the government’s responsibility for the crisis, influence people’s responses, and we examine the mediating role of experienced positive and negative affect on this response. To our knowledge, this is the first study to investigate humorous crisis communication among public sector organizations.

## Theoretical Background

The following section first provides brief background information on crises and crisis communication in general. Then relevant literature regarding the role of attributed crisis responsibility, emotions, and humor in crisis communication on social media is described to later derive our study hypotheses from.

### Crises, Crisis Communication, and Situational Crisis Communication Theory (SCCT)

A crisis can be described as “an event that is an unpredictable, major threat that can have a negative effect on the organization, industry, or stakeholders if handled improperly” (Coombs 1999, p. 2). Furthermore, it is characterized by “ambiguity of cause, effect, and means of resolution” (Pearson and Clair 1998, p. 60). Managing a crisis requires an organization to interact with the stakeholders to mitigate negative consequences, and crisis communication is the “verbal, visual, and/or written interaction between the organization and its stakeholders (often through the media) prior to, during and after a negative occurrence” (Fearn-Banks 2002, p. 480). These communication processes aim to provide information and support to stakeholders or the public, but they also help the organization manage and repair its reputation and legitimacy (Sturges 1994). Coombs’ (2007) situational crisis communication theory (SCCT) offers empirically based guidelines of how organizations can minimize reputation damage. According to SCCT, central factors affecting stakeholders’ emotional, attitudinal, and behavioral responses to a crisis are the degree to which the organization is responsible for the crisis (crisis responsibility) and the organization’s crisis response strategy, which should match its crisis responsibility (Coombs 2007; Ma and Zhan 2016).

### Attributed Crisis Responsibility Has Negative Consequences

According to Coombs (1998, p. 180), crisis responsibility can be defined as “the degree to which stakeholders blame the organization for a crisis event.”. When the organization is seen as a victim of the crisis, it is minimally held accountable (e.g., in the case of natural hazard-related disasters or rumors). However, a preventable crisis, such as a human-error accident, leads to strong attributions of crisis responsibility (Lee 2004). When the attributed crisis responsibility is high, several negative consequences follow: (1) *Reduced trust and reputation.* Several studies have found a negative effect of crisis responsibility on trust and organizational reputation (Coombs and Holladay 2007; Kim and Niederdeppe 2013; Ma and Zhan 2016) in private corporations and in public organizations. For instance, Bakker et al. (2018) revealed that high attribution of crisis responsibility leads to reduced trust in the local government, whereas trust in the local government scored higher when the government was not made accountable for the crisis. (2) *Damaging behavior.* When the attributed crisis responsibility is high, stakeholders are more willing to engage in damaging behavior, for example boycotting an organization (Coombs and Holladay 2007; Grappi and Romani 2015) or spreading negative word-of-mouth, which further damages the organization’s reputation (McDonald et al. 2010). (3) *Potential decrease in supportive behavior.* Studies show that the level of crisis responsibility does not influence stakeholders’ willingness to follow the local government’s advice (Bakker et al. 2018) or to share the organization’s crisis communication with others (secondary crisis communication, Utz et al. 2013). However, since high crisis responsibility has a negative effect on trust, it is conceivable that high crisis responsibility lowers people’s willingness to behave in ways that support the organization. (4) *Negative emotions.* The attribution of high crisis responsibility generally leads people to have more negative affect, especially more feelings of anger toward an organization (McDonald et al. 2010; Kim and Niederdeppe 2013; Utz et al. 2013), and it decreases people’s positive affect, such as joy or sympathy for an organization (Coombs and Holladay 2005; McDonald et al. 2010).

### Emotions Partially Mediate the Effect of Crisis Responsibility

Emotions play an important role in explaining the effectiveness of crisis communication (Coombs and Holladay 2005), since emotions function as a type of information processing system that helps people figure out how to view and respond to a specific situation (Loewenstein et al. 2001). Several studies suggest that emotions partially mediate between attributed crisis responsibility and people’s trust in an organization and behavioral intentions. On the one hand, having a negative affect damages the relationship between a stakeholder and an organization by reducing organizational trust and reputation (Choi and Lin 2009; Wang and Wanjek 2018); it also leads to more negative word-of-mouth comments and intentions to boycott an organization (Utz et al. 2013). On the other hand, positive affect, such as sympathy, improves one’s attitudinal and behavioral responses toward an organization (Kim and Niederdeppe 2013; Grappi and Romani 2015).

### Does Humorous Framing of Crisis Response Strategies Reduce Negative Emotions and Elicit Positive Emotions?

Several authors have suggested that using humor in crisis response strategies can decrease negative affect and increase positive affect (Vigsø 2013; Kim et al. 2016). The use of humor is expected to enhance stakeholders’ psychological coping mechanisms in crisis situations (Fredrickson et al. 2003) because humor can work as a buffer, lowering stress levels and the intensity of and the focus on negative emotions, such as fear and anger. Instead, it elicits feelings of happiness or cheerfulness (Fredrickson et al. 2003; Martin 2007). These positive emotions then broaden people’s thinking, leading to a more flexible and open-minded interpretation of a situation (Gulas and Weinberger 2006). Along that line of thinking, three decades of advertising research and even studies on ethically sensitive and threatening topics, such as climate change (Skurka et al. 2018), indeed reveal that humor influences emotional and cognitive processes, positively enhancing stakeholders’ attitudinal and behavioral reactions toward an organization (Eisend 2009, 2011). It leads to decreased counter-arguing and increased attention, liking of the source, purchase intentions, and generally to a more positive attitude toward a brand (Nabi et al. 2007; Eisend 2009).

According to these findings, humor might be useful in crisis communication. Kim et al. (2016) found that humorous crisis communication strategies on social media, such as a self-mocking and mocking-the-accuser strategy, cause recipients to have a more positive attitude toward an organization than when rational, non-humorous strategies are used. Furthermore, several case studies have verified the positive impact of humor in crisis communication on the relationship between an organization and its stakeholders during or after a crisis (Vigsø 2013). In line with SCCT (Coombs 2007), humorously framed crisis responses should match the organization’s crisis responsibility. Xiao et al. (2018) revealed that using humorously framed crisis responses on social media is less appropriate when the organization is responsible for a crisis, whereas in the case of a rumor (that is, low crisis responsibility) it is more effective.

Nevertheless, humor may reduce source credibility (Eisend 2009), which could reduce its positive effect on stakeholders’ trust in an organization (Mayer et al. 1995). Furthermore, humor can trivialize the perceived seriousness of a topic (that is, a crisis), making stakeholders less willing to perform the supportive behaviors proposed during a crisis (Fraustino and Ma 2015). Hence, humor is inappropriate in serious crises, as it reflects a lack of concern and empathy for the circumstances and decreases the perceived sincerity of an organization (Vigsø 2013; Xiao et al. 2018). However, it might be an effective tool in low-severity crises or in a paracrisis, defined as publicly visible crisis threats and accusations against an organization (Coombs and Holladay 2012). Especially on social media, where informal language is preferred, a humorously framed crisis response to a low-severity crisis could result in greater trust and contribute to the acceptance of the crisis response (Nabi et al. 2007; Kelleher 2009).

### The Present Study

Humorous crisis communication on social media might be useful in low-severity crises. By reducing negative affect and eliciting positive affect, humorous crisis communication might have positive effects on people’s trust in an organization, their view of the organization’s reputation, and their behavioral intentions. In this study, using an experimental research design, we investigated how people respond to humor in a local government’s crisis communication (presented as tweets, a typical social media format) depending on the government’s crisis responsibility. We chose trust as the central outcome variable, as the value of information given by governments is strongly reduced if they are not trusted (Steelman and McCaffrey 2013). This, in turn, can significantly influence citizens’ behavior in crises (Rubin et al. 2009).

Considering the damaging effect of attributed crisis responsibility for organizations (Coombs 2007), we postulate that in low-severity crises, high (versus low) crisis responsibility leads to less trust in the local government (H1a), more negative affect (H1b), and less positive affect (H1c). With regard to the influential role of emotions in explaining the effectiveness of crisis communication (Choi and Lin 2009), we expect that the effect of crisis responsibility on trust is mediated by the experienced positive or negative affect (H1d).

Considering that humor positively influences people’s emotions and stakeholders’ relationships with organizations (Eisend 2009, 2011; Kim et al. 2016), our second hypotheses state that humorously (versus rationally) framed crisis communication leads to more trust in the local government (H2a), less negative affect (H2b), and more positive affect (H2c). Moreover, based on findings by Eisend (2009, 2011), we expect that the effect of humorously framed crisis communication on trust is mediated by experienced positive and negative affect (H2d). Another question is whether the influence of humorously framed crisis communication on trust in the local government and on positive and negative affect is dependent upon whether the local government is responsible for the low-severity crisis (RQ1), since, to be most effective, crisis response strategies should match the organization’s crisis responsibility (Coombs 2007).

**Fig. 1:**
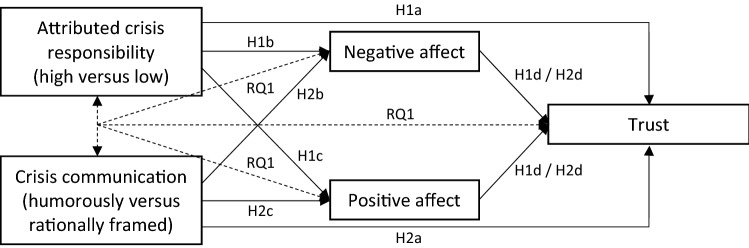
Research design illustrating the hypotheses H1a−H1d and H2a−H2d and the research question RQ1

**Fig. 2:**
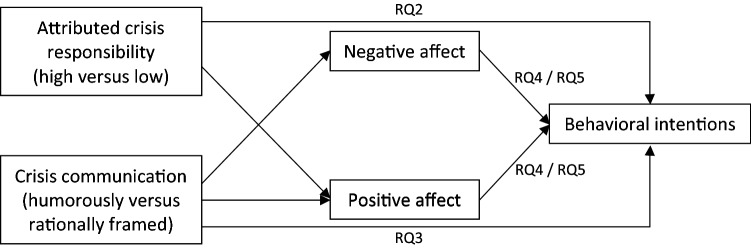
Research design illustrating the research questions RQ2−RQ5

Considering the influence of crisis responsibility and the framing of crisis responses on stakeholders’ different types of actions (Coombs 2007; Eisend 2009, 2011), we assume that crisis responsibility and the humorous framing of crisis response strategies influence people’s behavioral intentions toward an organization, but clear evidence for this is lacking, especially for behavioral intentions indicating that crisis communication was successful, such as people’s willingness to follow advice (Bakker et al. 2018) or secondary crisis communication (Utz et al. 2013). For this reason, we decided to examine exploratorily whether key actions—namely, people’s willingness to follow the local government’s advice, their willingness to seek information from the local government’s communication channels in crisis situations, and their secondary crisis communication—are influenced by the local government’s crisis responsibility (RQ2) and the framing of the crisis response strategy (RQ3). In addition, based on the conceptual model of SCCT, we wondered whether experienced positive or negative affect partially explain the effect that crisis responsibility (RQ4) and framing of the crisis response strategy (RQ5) have on people’s behavioral intentions. Our research design is visualized in Fig. 1 for H1, H2, and RQ1 and in Fig. 2 for RQ2−RQ5, respectively.

## Methodology

This section presents the methodology used in this study, including information on the pre-studies conducted to test study materials, the participants and their recruitment, the study design and procedure, and the materials and measures. Additional information on these contents can be found in the online supplement (including instructions and measures).^1^

### Pre-Studies

Two pre-studies were conducted to select the stimulus materials for the main study. In pre-study I, 10 participants (100% female) between 19 and 56 years old (*M*_age_= 25.70, *SD*_age_ = 10.75) were presented with nine different fictive scenarios (randomly ordered) describing crises in local governments’ areas of responsibility. Participants read two versions of each scenario, one where the local government was responsible for the crisis and one where it was not. After reading each scenario, participants rated the attributed crisis responsibility of the local government on a seven-point scale (a translated and adapted version of Lee 2004). A series of *t*-tests was carried out to check whether the local government was indeed considered responsible for the crisis in the respective scenarios.

In pre-study II, 13 participants (92% female) between 20 and 58 years old (*M*_age_ = 27.62, *SD*_age_ = 12.88) saw 32 fictive Twitter tweets posted by local governments in response to one of nine fictive crisis situations. Each post was preceded by a brief description of the background of the fictive crisis to which the post was related. Information on crisis responsibility was not given to avoid possible interaction effects between the government’s responsibility and the framing of the crisis response strategies (Xiao et al. 2018). Half of the tweets were humorously framed, while the other half were rationally formulated. Using an adapted version of Nabi et al.’s (2007) humor questionnaire, participants rated the perceived humor of each tweet on a seven-point scale. By using a series of *t*-tests or Wilcoxon signed-rank tests (if the assumption of normal distribution was violated) we compared whether humorous tweets were indeed perceived as funnier than rational ones. The three tweets perceived as the funniest, their rational equivalents and their respective scenarios were included in the main study.

### Participants

The a priori power analysis indicated that a sample size of 225 would be sufficient to detect significant effects in the planned parallel mediation models with a power of 0.80, an alpha of 0.05 and a medium effect size of *r* = |0.30|, which could be roughly expected following existing findings (Choi and Lin 2009; Eisend 2009; Kim and Niederdeppe 2013). Participants were recruited via two German online panels—PsyWeb^2^ and Fire Feedback^3^—and different social media platforms ensuring participants’ familiarity with online communication platforms, such as Twitter. Participants had to be at least 18 years old and speak German as their mother tongue or second language. Participation was voluntary, anonymous, and incentivized by the possibility of receiving a research report after data collection. Of 936 participants who started the questionnaire, 542 completed the full survey. Data from 25 participants were excluded from the analysis because they did not speak German as their mother tongue or second language (3), they did not allow the use of their data (17), or they did not answer the questionnaires seriously (5), as identified by an unrealistic short overall survey response time under seven minutes and a wrong answer in the attention check (cf. Meade and Craig 2012). Thus, the final sample consisted of 517 participants (272 male, 236 female, 4 diverse, 5 not specified). Ages ranged between 18 and 74 years old (*M* = 43.67, *SD* = 15.97). Participants’ highest completed educational levels were compulsory basic secondary schooling (4.4% of participants), a general certificate of secondary education (13.0%), a general or specialized university entrance qualification (30.4%), a degree from a university or college of higher education (47.8%), or a doctorate (3.3%); the remainder (1.2%) had an unspecified school-leaving qualification. Of the participants, 9.1% pursued a career in local government, 5.4% in crisis management, 34.8% in fields of fire safety, rescue services, or disaster control, and 3.9% in the police force or similar security services; 57.8% worked in none of these areas.^4^ The final dataset and data from excluded participants did not differ significantly in central characteristics, except for sense of humor, *Z* = 1,660.5, *p* = 0.048.

### Study Design and Procedure

This study was preregistered on the open science platform As Predicted^5^ and approved by the ethics board of the University of Münster’s Faculty 7, Psychology & Sports Science (ID 2021-41-MT). Data were collected on the online platform EFS Survey (Questback GmbH, 2017) between 25 May 2021 and 18 June 2021. The completion of the study took about 13 min on average (median = 12.40; *M* = 13.35 min, *SD* = 5.69). This study used a 2 (crisis responsibility: high versus low) × 2 (crisis response strategy: humorous versus rational) between-subjects design, resulting in four experimental conditions.^6^ All instructions, manipulations, and questionnaires were written in German. Participants were informed that the purpose of the 15-min study was to find out more about the effectiveness of local governments’ crisis communication. No information on humorous crisis communication was given at the beginning.

### Materials and Measures

The following section provides detailed information on the study materials and measures. Examples for the materials and scales used are given.

#### Crisis Scenarios and Tweets

Fictional crisis scenarios were used to avoid interference from local governments’ crisis histories and negative pre-crisis reputations that could have influenced the effectiveness of crisis communication (Coombs 2007). All crisis scenarios started with a short introduction where participants were asked to imagine that they lived in a fictional city; then they were given a description of a low-severity crisis having a certain importance for the city’s residents. It was decided to choose scenarios that can basically occur anywhere and affect anyone so that the participants could easily relate to the situations described. The scenarios were about a road salt shortage after an intense snowfall, a dysfunctional speed camera, and dysfunctional ticket machines in the local transport system. At the end of each scenario, information on the crisis’ cause was given. The local government could be either held responsible or not responsible for the crisis.

**Fig. 3:**
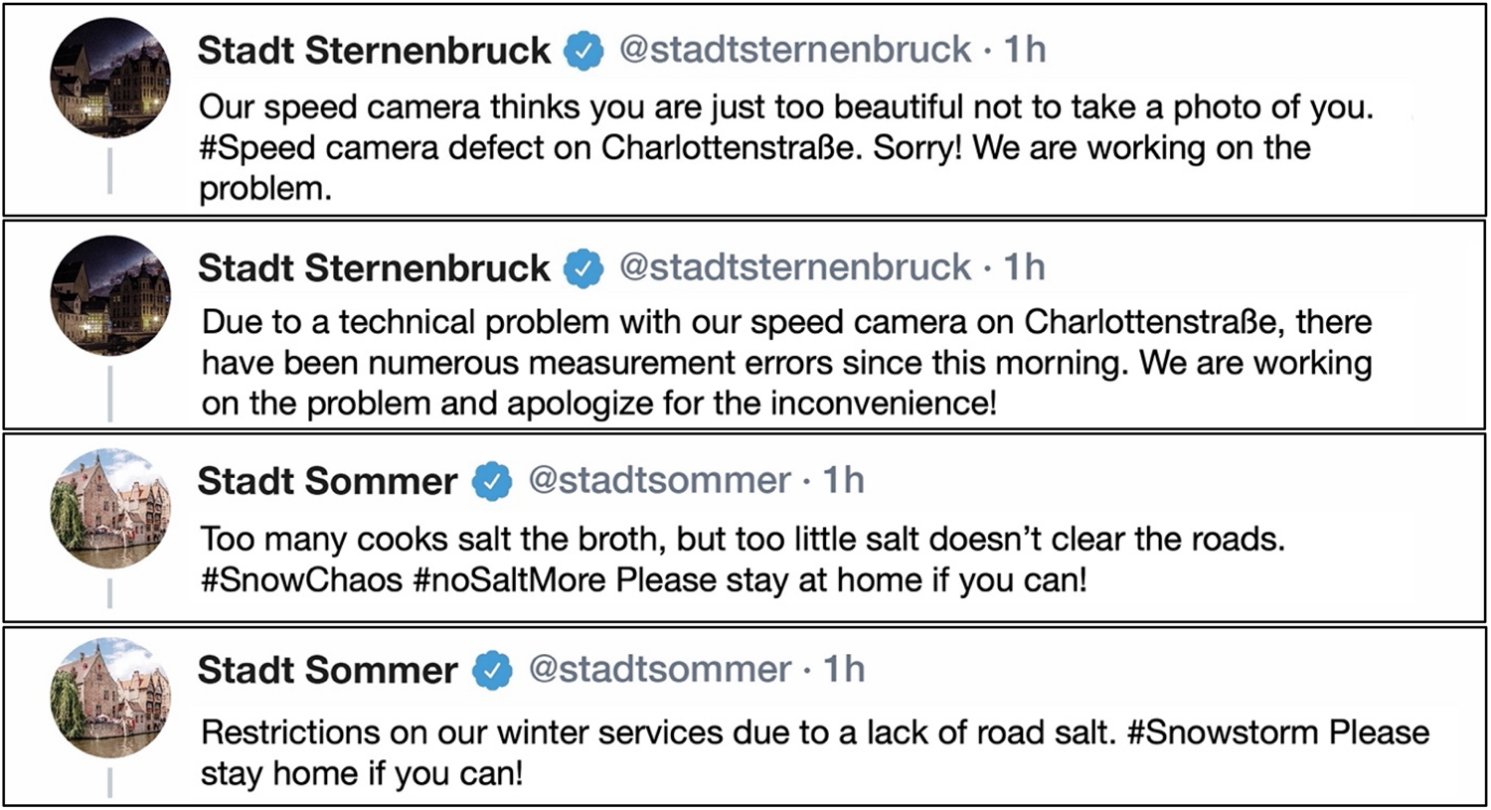
Examples of two humorous tweets and their corresponding rational tweets

In the respective tweets, the local government informed the public about the situation either in a rational or in a humorous way. All tweets used a subtype of the rebuilding response strategy (e.g., apology) (see Coombs 2007), and the same subtype was used in both versions of the tweet. No further details were given in the tweets concerning the cause of the crisis. Figure 3 illustrates examples of the tweets. We used a visualization based on Twitter as exemplary social media platform as Twitter is often linked to official German news sites and can be accessed easily without a user account.

#### Measures

The scales we used to measure participants’ positive and negative affect, trust in the local government, and behavioral intentions, including willingness to follow advice, secondary crisis communication, and willingness to seek information, are depicted in Table 1. To check whether the manipulation worked, we measured the crisis responsibility attributed to the local government and the perceived humor of the tweets. Furthermore, we checked attention by including one item within the questionnaire measuring behavioral intentions; this item asked participants to select the specific answer “Agree” for quality control reasons (Meade and Craig 2012). We decided to measure two control variables—namely, participants’ propensity to trust and sense of humor—because individual differences in these two dispositions could have influenced the effectiveness of the study manipulation. More precisely, disposition to trust can significantly influence the perceived trustworthiness of others (Mayer et al. 1995; Beierlein et al. 2014), whereas interindividual differences in senses of humor can explain why people differ in their sensitivity to humorous cues, in how much they like other humorous people and social interactions, and in expressions of mirthfulness and laughter (Svebak 2010).

**Table 1:** Measures of affect, trust, behavioral intentions, the manipulation check, and control variables

Construct	Example item	Origin scale	Reliability	Measurement scale
Positive and negative affect^a^	Active	PANAS Short Form (I-PANAS-SF) by Thompson (2007); German translation by Randler and Weber (2015)	Acceptable to good	Five-point scale from 1 (not at all) to 5 (extremely)
Trust in the local government^a^	I can trust the local government.	Hirschfeld and Thielsch (2022)	Good	Seven-point scale from 1 (strongly disagree) to 7 (strongly agree)
Liking of the tweet^a^	I like the way the local government communicates on Twitter during the crisis.	Developed for this study	–	Seven-point scale from 1 (strongly disagree) to 7 (strongly agree)
Willingness to follow advice^b^	I would follow the local government’s advice in a crisis.	Developed for this study	Poor	Seven-point scale from 1 (totally disagree) to 7 (totally agree)
Secondary crisis communication^b^	I would tell family and friends about the local government’s tweet.	Schultz et al. (2011)	Poor	Seven-point scale from 1 (totally disagree) to 7 (totally agree)
Willingness to seek information^b^	I would visit the local government’s website in a crisis situation to get more information about the crisis.	Kim and Niederdeppe (2013)	Poor	Seven-point scale from 1 (totally disagree) to 7 (totally agree)
Attributed crisis responsibility^a^	How much responsibility does the local government bear for the crisis?	Lee (2004)	Excellent	Seven-point scale from 1 (not at all to be blamed / not at all responsible) to 7 (absolutely to be blamed / totally responsible)
Perceived humor^a^	Not funny / funny	Nabi et al. (2007)	Excellent	Seven-point scale from 1 (strongly disagree) to 7 (strongly agree)
Propensity to trust^c^	I am convinced that most people have good intentions.	*Kurzskala Interpersonales Vertrauen* (*KUSIV3*; English: Interpersonal Trust Short Scale) by Beierlein et al. (2014)	Acceptable	Five-point scale from 1 (do not agree at all) to 5 (completely agree)
Sense of humor^d^	Persons who are always out to be funny are really irresponsible types not to be relied upon.	Ultra-Short Sense of Humor Questionnaire-3 (Ultra-short SHQ-3) by Svebak et al. (2004)	–^e^	Four-point scale from 1 (very sluggishly or not at all) to 4 (very easily or yes indeed).

If necessary, the scales were translated to German and/or the wording of the original scales was adapted to the respective scenarios.

^a^Mean values for each participant over all scenarios were calculated.

^b^Due to poor internal consistencies, we decided not to calculate mean values for the three behavioral intention subscales over all scenarios, and each item was analyzed on its own.

^c^Average interpersonal trust was calculated for every participant.

^d^An overall index of sense of humor was calculated by summing the three items.

^e^Due to missing item homogeneity, Cronbach’s alpha could not be calculated.

## Results

In this part, the results of the manipulation checks, the hypotheses testing, and exploratory analyses are presented. Detailed information on descriptive data and additional statistical analyses can be found in the online supplement.^7^

### Manipulation Checks: Attributed Crisis Responsibility and Perceived Humor

Two ANOVAs suggested that the experimental manipulations worked very well. Local governments were held more responsible for the crisis in the high-responsibility crisis scenarios (*M* = 5.61, *SD* = 0.94) than in the low-responsibility ones (*M* = 3.44, *SD* = 1.26), *F*(1, 513) = 495.90, *p* < 0.001, η_p_^2^ = 0.49, and humorously framed crisis response strategies were perceived as funnier (*M* = 4.25, *SD* = 1.70) than the rational ones (*M* = 2.23, *SD* = 1.50), *F*(1, 513) = 356.25, *p* < 0.001, η_p_^2^ = 0.41.

### H1, H2, and RQ1: The Effect of Crisis Responsibility and Humorous Framing on Trust, Negative Affect, and Positive Affect

Hypotheses about the main effect of crisis responsibility (H1a−H1c) and humorous framing (H2a−H2c) and their interaction effect (RQ1) on trust, positive affect, and negative affect were tested with a MANOVA. Here, Pillai’s trace indicated significant effects of crisis responsibility, *V* = 0.04, *F*(3, 511) = 6.76, *p* < 0.001, η_p_^2^ = 0.04 (1 – β = 1.00), humorous framing, *V* = 0.02, *F*(3, 511) = 3.81, *p* = 0.010, η_p_^2^ = 0.02 (1 – β = 0.95), and a crisis responsibility × humorous framing interaction effect, *V* = 0.02, *F*(3, 511) = 2.97, *p* = 0.032, η_p_^2^ = 0.02 (1 – β = 0.95).

The follow-up ANOVA for trust yielded a significant result for crisis responsibility, *F*(1, 513) = 9.57, *p* = 0.002, η_p_^2^ = 0.02 (1 – β = 0.90), suggesting that trust in the government was higher when the crisis responsibility was low (*M* = 4.78, *SD* = 0.95) than when it was high (*M* = 4.51, *SD* = 1.02). Furthermore, a significant main effect of framing was found, *F*(1, 513) = 5.67, *p* = 0.018, η_p_^2^ = 0.01 (1 – β = 0.63), suggesting that trust in the local government was higher with the rational framing (*M* = 4.75, *SD* = 1.02) than the humorous framing (*M* = 4.53, *SD* = 0.96). Yet, there was no significant crisis responsibility × framing interaction effect on trust in the local government, *F*(1, 513) = 3.55, *p* = 0.060, η_p_^2^ = 0.01.

The follow-up ANOVA for negative affect showed a significant effect for crisis responsibility, *F*(1, 513) = 12.14, *p* < 0.001, η_p_^2^ = 0.02 (1 – β = 0.90), suggesting that negative affect was higher when the crisis responsibility was high (*M* = 1.63, *SD* = 0.46) than when it was low (*M* = 1.49; *SD* = 0.44). Neither the main effect of framing, *F*(1, 513) = 0.53, *p* = 0.467, η_p_^2^ < 0.01, nor the interaction effect on negative affect, *F*(1, 513) =1.80, *p* = 0.180, η_p_^2^ < 0.01, were significant.

The follow-up ANOVA for positive affect revealed that crisis responsibility did not significantly influence positive affect, *F*(1, 513) = 3.41, *p* = 0.065, η_p_^2^ = 0.01. However, it indicated a significant effect for framing, *F*(1, 513) = 5.96, *p* = 0.015, η_p_^2^ = 0.01 (1 – β = 0.63), suggesting that positive affect was lower with the humorous framing (*M* = 2.17; *SD* = 0.83) than with the rational framing (*M* = 2.35; *SD* = 0.82). The interaction effect on positive affect was not significant either, *F*(1, 513) = 0.35, *p* =0.553, η_p_^2^ < 0.01. All effects found in the MANOVA and its follow-up ANOVAs are small according to Cohen (1988), who defined η^2^ < 0.06 as small effects.

### H1d and H2d: The Mediating Role of Experienced Positive or Negative Affect on Trust

**Fig. 4:**
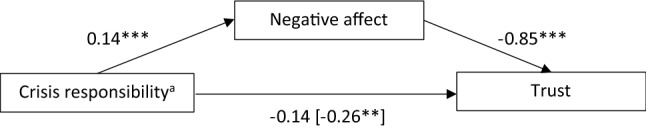
Negative affect mediates the effect of crisis responsibility on trust. Representation of the unstandardized direct effects and the total effect (in brackets). ^a^0 = low crisis responsibility, 1 = high crisis responsibility. **p < 0.01. ***p < 0.001.

Due to missing effects of crisis responsibility on positive affect and humorous framing on negative affect, we subsequently conducted simple instead of parallel mediation analyses. The first mediation analysis tested whether negative affect mediates the relationship between crisis responsibility and trust (H1d). We found a significant indirect effect of crisis responsibility mediated by negative affect, *ab* = −0.12, 95% CI [−0.20, −0.05], *p* < 0.001 (1 – β = 0.93). High crisis responsibility led to higher negative affect, resulting in lower trust. The other path coefficients are presented in Fig. 4.

**Fig. 5:**
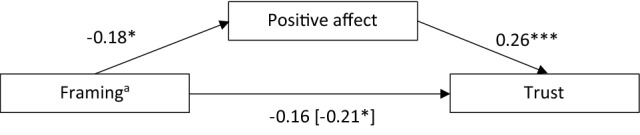
Positive affect mediates the effect of framing on trust. Representation of the unstandardized direct effects and the total effect (in brackets). ^a^0 = rational framing, 1 = humorous framing. *p < 0.05. ***p < 0.001.

The second mediation analysis investigated the mediating role of positive affect on the relationship between framing and trust (H2d). It revealed a significant indirect effect of framing mediated by positive affect, *ab* = −0.05, 95% CI [−0.10, −0.01], *p* = 0.002 (1 – β = 0.70). Participants seeing the rational framings experienced more positive affect, and, in turn, participants who experienced more positive affect gave higher ratings of trust. The other path coefficients are presented in Fig. 5.

### RQ2 and RQ3: The Effect of Crisis Responsibility and Framing on Behavioral Intentions

The main effect of crisis responsibility (RQ2) and humorous framing (RQ3) on variables measuring behavioral intentions were tested with a MANOVA. Here, Pillai’s trace indicated a significant effect of crisis responsibility, *V* = 0.04, *F*(6, 508) = 3.39, *p* = 0.003, η_p_^2^ = 0.04, but not for framing, *V* = 0.02, *F*(6, 508) = 1.43, *p* = 0.201, η_p_^2^ = 0.02, nor for the crisis responsibility × framing interaction effect, *V* = 0.01, *F*(6, 508) = 0.74, *p* = 0.619, η_p_^2^ = 0.01.

When the local government’s crisis responsibility was high, participants showed less willingness to follow the local government’s advice (high: *M* = 4.90, *SD* = 1.04; low: *M* = 5.15, *SD* = 1.04), they were less willing to follow local government’s advice as a matter of principle (high: *M* = 5.58, *SD* = 1.35; low: *M* = 5.89, *SD* = 1.25), they were less willing to ask family and friends to follow advice (high: *M* = 4.60, *SD* = 1.24; low: *M* = 4.83, *SD* = 1.21), and they were less willing to seek information on the local government’s website (high: *M* = 5.21, *SD* = 1.23; low: *M* = 5.55, *SD* = 1.14). Moreover, the willingness to ask family and friends to follow advice was lower when a humorous (*M* = 4.59, *SD* = 1.26) rather than a rational framing was used (*M* = 4.83, *SD* = 1.19). All significant effects found in the MANOVA and its follow-up ANOVAs are small according to Cohen’s (1988) conventions.

### RQ4 and RQ5: The Mediating Role of Positive and Negative Affect on Behavioral Intentions

We tested whether negative or positive affect mediates the relationship between crisis responsibility or humorous framing and behavioral intentions (RQ4 and RQ5). Similar to the mediation analyses above, simple instead of parallel mediation analyses were conducted. Mediation analyses were only conducted for the behavioral intention variables that were significantly influenced by crisis responsibility or framing. All results of the mediation analyses can be found in Table 2; they show that affect fully mediated the effect of crisis responsibility on willingness to follow advice and secondary crisis communication but not on willingness to seek further information. Affect partially mediated the effect of framing on secondary crisis communication.

**Table 2:** Mediation effect of crisis responsibility or framing on behavioral intentions by negative or positive affect, respectively

Factor	Variable	*a*	*b*	*c’*	*c*		*ab*		*p*
							95% CI		
						est.	*LL*	*UL*	
Crisis responsibility^a^	*Willingness to follow advice*								
	I would follow local government’s advice in a crisis.	0.14***	−0.61***	−0.16	−0.24*	−0.08***	−0.15	−0.04	< 0.001
	As a matter of principle, I would not follow advice from the local government in a crisis.^b^	0.14***	−0.18	−0.29*	−0.31**	−0.03	−0.08	0.00	0.144
	*Secondary crisis communication*								
	I would ask family and friends to follow the advice given by the local government in a crisis situation.	0.14***	−0.38**	−0.17	−0.23*	−0.05**	−0.11	−0.02	0.002
	*Willingness to seek information*								
	I would visit the local government’s website in a crisis situation to get more information about the crisis.	0.14***	−0.15	−0.32**	−0.34**	−0.02	−0.07	0.01	0.228
Framing^c^	*Secondary crisis communication*								
	I would ask family and friends to follow the advice given by the local government in a crisis situation.	−0.018*	0.24***	−0.019	−0.23*	−0.04	−0.10	−0.01	0.010

Low crisis responsibility was coded as 0 and high crisis responsibility was coded as 1. Rational framing was coded as 0 and humorous framing was coded as 1. All significance tests are two tailed. *a* = unstandardized effect of crisis responsibility on negative affect; *b* = unstandardized effect of negative affect on the respective behavioral intention variables adjusted for crisis responsibility; *c*’ = unstandardized direct effect of crisis responsibility on the respective behavioral intention variables adjusted for negative affect; *c* = unstandardized total effect; *ab* = indirect effect; CI = confidence interval; *LL* = lower limit; *UL* = upper limit.

^a^Negative affect was used as a mediator.

^b^In the interest of consistency, the reversed version of this item was used in calculations.

^c^Positive affect was used as a mediator.

*p < 0.05. **p < 0.01. ***p < 0.00.

### Exploratory Analyses

In four exploratory analyses, we examined (1) further predictors of trust; (2) the moderating role of participants’ demographics and characteristics; (3) the moderating effect of the crisis scenarios themselves; and (4) participants’ liking of the tweets depending on both experimental factors. Additionally, four exploratory analyses were examined.^8^ The results of the first exploratory analysis can be found in Table 3; these results showed that both control variables and some demographic variables contributed to the predication of trust. The second and third exploratory analyses indicated that some characteristics (e.g., disposition to trust) and the scenarios themselves moderated the effect of crisis responsibility and framing on trust. The forth exploratory analysis yielded a significant effect of framing, *F*(1, 513) = 8.39, *p* = 0.004, η_p_^2^ = 0.02, and indicated that participants preferred rational framing (*M* = 4.42, *SD* = 1.23) rather than humorous framing (*M* = 4.07, *SD* = 1.44).

**Table 3:** Results of the hierarchical regression analysis predicting trust

	1			2			3		
	*b*	*SE b*	*β*	*b*	*SE b*	*β*	*b*	*SE b*	*β*
(Intercept)	4.874***	0.074	0.000	4.912***	0.235	0.000	4.988***	0.228	0.000
Crisis responsibility^a^	−0.262**	0.086	−0.132	−0.273**	0.087	−0.137	−0.279***	0.084	−0.140
Framing^b^	−0.208*	0.087	−0.105	−0.216*	0.087	−0.109	−0.233**	0.085	−0.117
Age				−0.008*	0.003	−0.124	−0.007*	0.003	−0.109
*Sex*^c^									
Female				−0.044	0.110	−0.022	−0.028	0.106	−0.014
Diverse				0.713	0.498	0.063	0.816	0.484	0.072
*Education*^d^									
GCSE O-levels				−0.033	0.238	−0.011	−0.068	0.231	−0.023
Specialized A-levels				−0.303	0.248	−0.089	−0.385	0.241	−0.112
A-levels				0.044	0.231	0.018	−0.005	0.224	−0.002
College of higher education degree				0.128	0.226	0.052	0.020	0.220	0.008
University degree				−0.102	0.225	−0.046	−0.202	0.218	−0.090
Doctorate				0.218	0.317	0.039	0.067	0.309	0.012
Another certificate				0.155	0.452	0.017	0.083	0.438	0.009
*Working area*									
Local government^e^				0.162	0.163	0.047	0.205	0.158	0.059
Crisis management^e^				−0.400	0.210	−0.091	−0.369	0.203	−0.084
Emergency response^e^				−0.011	0.123	−0.005	−0.020	0.120	−0.010
*Police*^e^				0.355	0.225	0.070	0.397	0.218	0.077
Disposition to trust^f^							0.279***	0.059	0.210
Sense of humor^f^							0.058*	0.028	0.094
*R*^2^	0.03			0.08			0.14		
Δ*R*^2^				0.05*			0.06***		

*N* = 517. All significance tests were two tailed.

^a^0 = low crisis responsibility, 1 = high crisis responsibility.

^b^0 = rational framing, 1 = humorous framing.

^c^Male was coded as reference category.

^d^Lower secondary school leaving certificate was coded as reference category.

^e^0 = no, 1 = yes.

^f^Age, disposition to trust, and sense of humor were centered.

*p < 0.05. **p < 0.01. ***p < 0.001.

## Discussion

Inspired by recent propositions that local governments and public authorities may want to use less formal crisis communication strategies on social media, this study offers first insights into the effectiveness of humorous crisis communication on social media by public sector organizations in low-severity crises. It further highlights once again the importance of crisis responsibility in crisis communication.

### Attributed Crisis Responsibility Evokes Negative Affect and Reduces Trust

According to our first hypothesis, high attributed crisis responsibility predicted a reduction in trust (H1a) and a rise in negative affect (H1b). This is in line with earlier findings (Coombs 2007; Choi and Lin 2009; Bakker et al. 2018). We also replicated the mediating role of experienced negative affect in explaining the decrease in trust (H1d; see Choi and Lin 2009; Kim and Niederdeppe 2013). However, a local government’s high crisis responsibility did not reduce or increase positive affect (H1c). This stands in contrast to the findings by Coombs and Holladay (2005), McDonald et al. (2010), and Kim and Niederdeppe (2013). Consequently, the mediating effect of experienced positive affect on the relationship between attributed crisis responsibility and trust found by past researchers (Kim and Niederdeppe 2013; Grappi and Romani 2015) did not occur in the present study (H1d).

The lack of correlation between crisis responsibility and positive affect might have occurred because the study’s opposing effects neutralized each other: For example, while positive emotions directed to the organization, such as sympathy, are negatively related with crisis responsibility, the positive emotions associated with one’s own situation in a crisis, such as relief, are partially positively correlated with crisis responsibility (McDonald et al. 2010).

### Humorous Crisis Communication Does Not Have a Beneficial Effect

Contrary to our second hypothesis, we did not find a positive effect of humor on trust (H2a) or on experienced emotions (H2b and H2c), such that emotions played no mediating role (H2d), even though the manipulation check showed that humorously framed crisis responses were indeed perceived as humorous. This pattern of results contradicts earlier studies in humor (Martin 2007) and advertising research (Eisend 2009, 2011), making clear that findings on humorous communication from these research areas cannot be transferred to a public sector’s online crisis communication. Our results also disagree with findings from private sector online crisis communication (Kim et al. 2016), but they are in line with more recent research by Honisch and Más Manchón (2020) and Xiao et al. (2018), who advised against using humorously framed crisis response strategies on social media, as they resulted in lower levels of organizational reputation compared with more rational ones.

The independence of perceived humor and emotional reactions found in our study can be explained by the benign violation theory of humor (Warren and McGraw 2015). Humor can violate a person’s well-being, normative beliefs, or identity, and the degree to which the violation is acceptable explains the extent to which negative emotional reactions are evoked (Warren and McGraw 2015). Especially in a crisis, humor can be perceived as inacceptable (Liu et al. 2013), thus evoking less positive and more negative emotions. Several participants indeed commented at the end of the study that they considered humor inappropriate in a crisis. The exploratory analysis also confirmed that participants liked the humorous tweet less than the rational one. However, it is interesting to recognize that only positive affect, not negative affect, was significantly influenced by the framing in this study. The overall low level of negative affect in the present sample might explain this.

Furthermore, several moderators might elucidate the results. For instance, the exploratory analysis revealed that individual differences in disposition to trust moderated the effect of humorous framing on trust. Additional potential moderators are participants’ prior image of local governments (Kim et al. 2016), which may have influenced perceived message credibility (Nabi et al. 2007), the social media culture itself (Kelleher 2009; Kim et al. 2016), and the quality of participants’ prior relationship with real local governments (Jahng and Hong 2017). Moreover, the significant scenario × framing interaction effect on trust in our exploratory analysis indicates that the effectiveness of humorously framed crisis response strategies depended on the specific context’s characteristics. One important contextual factor might be the individuals’ level of involvement with the crisis, which could affect the effectiveness of humorous communication (Yoon and Tinkham 2013).

Interestingly, we did not find a significant interaction effect of crisis responsibility and humorously framed crisis response strategies on trust in local government, positive affect, or negative affect. This contrasts with some basic assumptions of SCCT (Coombs 2007) and findings by Xiao et al. (2018), which assume that crisis response strategies should match the organization’s crisis responsibility to protect the organizational image. However, our findings are in line with research by Honisch and Más Manchón (2020), who found that humorous crisis communication on social media seems to be ineffective regardless of an organization’s level of attributed crisis responsibility.

### Behavioral Intentions

In exploratory analyses, we found that behavioral intentions were predicted by attributed crisis responsibility but not by humor. In line with earlier research (McDonald et al. 2010; Grappi and Romani 2015), high attributed crisis responsibility led to a decrease in supportive behavior. Humorously framed crisis communication, however, did not influence behavioral intentions. This reflects recent ambiguous findings in humor research (see, e.g., Liu et al. 2013; Honisch and Más Manchón 2020) and illustrates that public authorities should use humor with caution.

### Limitations and Future Research

The experimental design is a key strength of our study. However, it imposes some limitations in terms of variables included and ecological validity, which, in turn, offer opportunities for future research. A first limitation is that we only tested the humorous framing of rebuilding response strategies using affiliative humor and rhyme. In terms of future research, it would be useful to extend current findings by examining the humorous framing of different crisis response strategies (Coombs 2007) and using different humor types (Martin et al. 2003). As we only used a visualization based on Twitter as exemplary social media platform, it might also be interesting to examine whether there are any platform-specific effects. Further, we only investigated experienced positive and negative affect as mediating variables. As specific emotions associated with crisis responsibility influence attitudes and behaviors toward an organization differently, future studies should investigate the effects of affect beyond their valence (Raghunathan et al. 2006). Additionally, the results of the exploratory analyses revealed the need to further examine other, potentially opposing mediating variables, such as cognitive mechanisms (e.g., perceived severity), which might influence participants’ evaluation of their relationship with the local government as well as their decision to act (Moyer-Gusé et al. 2011).

It is unclear to what extent intentions, as measured in our study, would translate into actual behavior in real-life scenarios. According to the literature, behavioral tendencies do not always lead to actual behavior (Baumeister et al. 2007). Thus, in addition to intention itself, it may be important here to consider in parallel other key variables related to individuals’ ability and confidence to act: For example, in a recent experiment on laypersons behavior in a low-severity crisis situation (the confrontation with an incipient fire in a work setting), self-efficacy beliefs were a significantly stronger predictor of behavior than intentions (Thielsch et al. 2021). However, some research in crisis communication indicates that behavioral intentions can be important drivers for actual behavior in crisis situations (Weyrich et al. 2020). Hence, future research should analyze the impact of humorous crisis communication in different crisis types on both short- and long-term crisis-related behavior in field experiments while at the same time considering other key variables such as self-efficacy.

We tested our experimental material in two pre-studies. Yet, the exploratory analysis revealed that the results were not independent of the specific scenario presented. On the one hand, this finding can be considered a study limitation, in that we failed to create low-severity crisis scenarios with the same level of perceived crisis severity. On the other hand, these findings offer promising avenues for future research. As already discussed, we see that contextual and individual factors (such as personal involvement) could strengthen or reduce the effects found.

Finally, one might criticize our use of SCCT (Coombs 2007) as a theoretical framework for investigating communication during a crisis, although results from this study clearly support its postulated structure. Coombs (2007) himself described SCCT as a post-crisis communication theory designed to repair an organization’s reputation after having addressed the physical and psychological threats of crisis victims. However, Olsson (2014, p. 117) opposed this by claiming that dividing crisis responses into ones that provide instructing information and ones that address reputational concerns is “somewhat artificial,” as an organization’s crisis communication and actions during the whole course of a crisis influence the relationship with the stakeholders. Scholars (e.g., Liu et al. 2012) have started to establish models specifying which crisis response strategies should be used in a specific phase of a crisis, but empirical research on critical response time in crisis scenarios is limited. It might be interesting to explore whether humorous online crisis communication works better after a crisis rather than during the incident and how knowledge on a local government’s crisis responsibility affects its effectiveness at that specific point in time.

### Practical Implications

Our core message for local governments’ crisis communication managers is as follows: If your local government is responsible for a crisis, do not use humorous framing in crisis communication on social media, even in low-severity crises. For humor to have a positive effect, crisis communication managers would have to accurately consider many factors, such as the specific characteristics of the crisis (e.g., crisis severity), the local government (e.g., prior image), and the target group (e.g., involvement). Additionally, the style of humor (Martin et al. 2003) should be chosen wisely. If crisis communication managers choose poorly, this can cause a loss in trust, induce negative emotions, and encourage the circulation of rumors (Park et al. 2012), which further damage citizens’ relationships with the local government. Because local governments are required to communicate quickly about crises via social media, crisis communication experts recommend preparing adequate crisis responses for different incidences in advance (Claeys and Opgenhaffen 2016). As such, it is questionable whether integrating humorous elements in formal online crisis communications might be effective, as humor is often found in the surprising and funny aspects of current circumstances, which cannot be known beforehand. Therefore, it might be helpful to use humorous elements only in post-crisis communication when the relationship between citizens and the local government has to be restored or improved.

## Conclusion

In summary, this study conducted the first experiment on the effectiveness of humor in local governments’ crisis communication via social media, its conditions, and working mechanisms. Our results mainly argue against the use of humor in online crisis communication because it negatively influences trust and positive affect. However, if the application is well planned and both the target group and framework conditions are suitable, humorous crisis communication on social media might be appropriate under specific circumstances (e.g., for post-crisis communication, low crisis involvement of the target group) in low-severity crises. We hope that the current study will motivate further investigation of possible moderating and mediating variables, such as quality of emotions and perceived crisis severity. Furthermore, the present research contributes to a growing body of evidence for the SCCT (Coombs 2007), suggesting that an organization’s level of crisis responsibility is one of the most important factors in crisis communication, as high levels of crisis responsibility negatively influence affect, trust, and behavioral intentions. If a public organization has caused a crisis, humorous communication cannot compensate for the perceived negative impact of this responsibility.

## Footnotes

1 https://doi.org/10.5281/zenodo.6983191

2 https://psyweb.uni-muenster.de

3 https://fire.uni-muenster.de

4 Multiple choice was allowed. Some participants worked in more than one job sector.

5 https://aspredicted.org/f8yy5.pdf

6 The four experimental groups consisted of 131, 131, 119, and 136 participants, respectively. They did not differ significantly in central characteristics, except for education, *p* = 0.016, and sense of humor, *F*(3, 513) = 3.91, *p* = 0.009, η_p_^2^ = 0.02.

7 https://doi.org/10.5281/zenodo.6983191

8 In the interest of parsimony, only relevant significant effects are reported for the exploratory analyses. Complete results and effect sizes are in the online supplement (https://doi.org/10.5281/zenodo.6983191).
